# Administration of selenomethionine in combination with serine benefits diabetes *via* gut microbiota

**DOI:** 10.3389/fmicb.2022.1007814

**Published:** 2022-10-12

**Authors:** Xiaoyan Cui, Jingqing Chen, Yuexi Yang

**Affiliations:** ^1^College of Animal Science and Technology, Yangzhou University, Yangzhou, China; ^2^Laboratory Animal Center of the Academy of Military Medical Sciences, Beijing, China; ^3^State Key Laboratory of Animal Nutrition, China Agricultural University, Beijing, China; ^4^School of Food Science and Biotechnology, Zhejiang Gongshang University, Hangzhou, China

**Keywords:** diabetes, microbe, selenium, selenomethionine, serine

## Abstract

Either selenium or serine could modulate glucose homeostasis, however, whether there are synergistic effects of selenium with serine on diabetes remains to be unknown. In the present study, eight male db/m mice were used as a control, and 24 male diabetic db/db mice were either orally gavaged with PBS, or with selenomethionine alone, or with both selenomethionine and serine, to investigate the effects of selenomethionine and serine on body weight and glucose level. Furthermore, intestinal microbiota composition was analyzed and fecal microbiota transplantation (FMT) was performed to explore whether microbes mediate the beneficial effects of selenomethionine and serine. The results showed that administration of selenomethionine decreased body weight, adipose tissue weight and serum glucose level in db/db diabetic mice. Importantly, administration of selenomethionine in combination with serine exerted better effects than selenomethionine alone did. Furthermore, a combined administration of selenomethionine and serine restored the microbial composition in diabetic mice. *Corynebacterium glutamicum*, *Bifidobacterium pseudolongum*, and *Aerococcus urinaeequi* were significantly decreased, whereas *Lactobacillus murinus* was increased in mice in the selenomethionine group and selenomethionine in combination with serine group, when compared with those in the db/db group. FMT decreased body weight and glucose level in db/db mice, further indicating that microbes play critical roles in the beneficial effects of selenomethionine and serine. Thus, we concluded that administration of selenomethionine in combination with serine benefits diabetes *via* gut microbes. Our results suggested that the synergic application of selenomethionine and serine could be potentially used for the treatment of diabetes.

## Introduction

Diabetes mellitus is a metabolic disease increasingly prevalent worldwide. It is characterized by long-term hyperglycemia which results in health problems for a long period. Although many drug portfolios are found to exert the effect of maintaining blood glucose concentrations, most of them are proved to have side effects. Consequently, although increasing new therapies have been successfully explored for diabetes treatment, the seek for novel treatment approaches which can better control glucose level and reduce related complications need to be continuously done.

Selenium, as an essential trace element, exerts critical roles in the maintenance of glucose homeostasis. A deficiency of selenium leads to increased glucose concentration in plasma in healthy rats and further elevated glucose concentration in diabetic individuals (Reddi and Bollineni, 2001). Supplementation with sodium selenite exerts insulin-like effects, and a high-dose of sodium selenite administrated acutely results in hypoglycemia (Becker et al., 1994; Stapleton, 2000). Importantly, selenomethionine, as an organic selenium source, was proved to involve in insulin secretion and glucose homeostasis (Zhao et al., 2021). However, no results have reported the *in vivo* beneficial effects of selenomethionine on diabetic individuals.

Either selenium or serine could modulate glucose homeostasis. Serine, as a metabolically indispensable amino acid, involved in purine and glutathione synthesis, one-carbon metabolic cycle and lipid metabolism (Zhou et al., 2017). Importantly, serine improves glucose tolerance and insulin sensitivity in high-fat diet-induced mice (Zhou et al., 2018). Interestingly, recent studies found that dietary serine had beneficial effects on nutritional status of selenium and selenoprotein activity (Long et al., 2021; He Y. et al., 2022; Zhou L. et al., 2022). Moreover, the synergistic effects of serine with selenium on the alleviation of oxidative stress were confirmed *in vivo* and *in vitro* (Wang et al., 2016; He L. et al., 2022). However, whether a combined administration of selenomethionine and serine has any effects on glucose homeostasis are not explored. It is hypothesized that administration of selenomethionine in combination with serine benefits diabetes *via* gut microbes. The present work has investigated both administration of selenomethionine alone and in combination with serine on body weight and glucose level in db/db diabetic mice. Furthermore, intestinal microbiota composition was analyzed and fecal microbiota transplantation (FMT) was performed to explore whether microbes mediate the beneficial effects of selenomethionine and serine.

## Materials and methods

### Animals and experimental design

Eight male db/m mice and 24 male db/db diabetic mice were purchased from Gene&Peace biotech Co., Ltd (Guangzhou, China). The db/m mice were orally gavaged with 0.2 mL PBS and used as control (db/m), while the db/db mice were either orally gavaged with 0.2 mL PBS (db/db), or with selenomethionine (0.16 mg/mL) dissolved in 0.2 mL PBS (SeMet), or with selenomethionine (0.16 mg/mL) and serine (0.20 g/mL) dissolved in 0.2 mL PBS (SeSer). The experiment lasted 21 days. During the experiment, all animals were free to access water and feed, and body weight (BW) was recorded. The study was approved by the Animal Care and Use Committee of China Agricultural University, and conformed to the Guide for the Care and Use of Laboratory Animals.

### Sample collection

On day 21, blood samples were collected from the retro-orbital sinus and then centrifuged at 2,500 *g* at 4°C for 10 min to collect serum. All mice were executed by cervical dislocation, and then inguinal fat, epididymal fat and liver were separated and weighted.

### Determination of serum glucose level

Serum glucose level was determined by using the commercially available kits (A154-1-1) from Nanjing Jiancheng Bioengineering Institute (Nanjing, China) according to manufacturers’ instruction.

### Gut microbiota profiling

Freshly collected feces were collected for DNA extraction. The V3–V4 region sequence of bacterial 16S rDNA gene was amplified and PCR reactions were performed as previously described (He L. et al., 2022). The sequencing was performed on the MiSeq Illumina platform, by Novogene Bioinformatics Technology Co., Ltd. (Tianjin, China). Then, the paired-end reads that obtained were assigned to samples based on their unique barcodes. By using USEARCH, the operational taxonomic units (OTUs) were obtained by clustering the high-quality clean tags. Subsequently, by using the Greengenes database and QIIME, representative OTUs were used for α- and β-diversity and principal coordinate analysis (PCoA).

### Fecal microbiota transplantation

The db/db diabetic mice were first orally gavaged with 0.2 mg ampicillin, 0.1 mg vancomycin, 0.2 mg neomycin, 0.2 mg streptomycin and 0.2 mg metronidazole dissolved in 0.2 mL PBS for 7 consecutive days as previously described (Zhou X. et al., 2022). Then, the db/db mice were gavaged with 5 mg freshly collected feces suspended in 0.2 mL PBS, either from mice in the db/db group or from those in the SeSer group. The feces were gavaged once daily for 7 consecutive days. Then, body weight and serum glucose level were determined on day 7 and 21.

### Statistical analysis

All data was analyzed using a t test or one-way ANOVA followed by S-N-K *post hoc* test (SPSS18.0). Data were presented as the mean ± SEM. Mean values were considered significantly different when *P* value < 0.05. Community composition of microbiota and diversity were analyzed using R software (version 3.3.1).

## Results

### Both administration of selenomethionine alone and in combination with serine decreased body weight and serum glucose level in db/db diabetic mice

**FIGURE 1:**
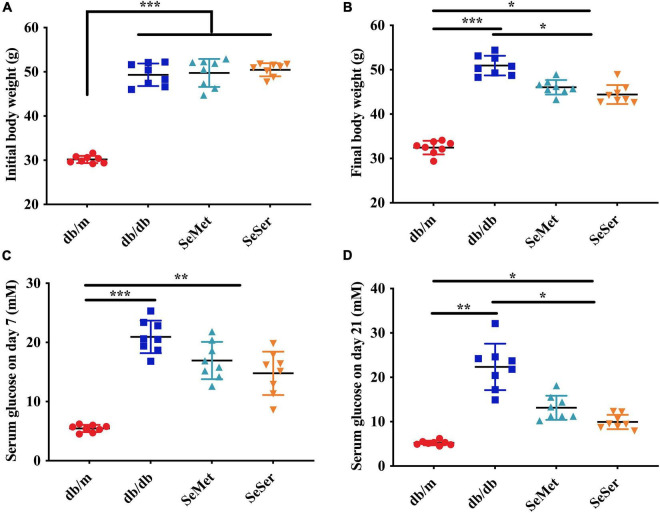
Administration of selenomethionine and serine decreased body weight and serum glucose level in db/db diabetic mice. **(A)** Initial body weight; **(B)** final body weight; **(C)** serum glucose level on day 7; **(D)** serum glucose level on day 21. Values are expressed as mean ± SEM, *n* = 8. *P < 0.05, **P < 0.01, ***P < 0.001.

As showed in Figure 1, db/db mice had significantly higher initial body weight than those of db/m mice (Figure 1A). At the end of the experiment, both administration of selenomethionine alone and in combination with serine significantly decreased final body weight of db/db mice, however, the body weight of these db/db mice remained to be significantly higher than those of db/m mice (Figure 1B). Both administration of selenomethionine alone and in combination with serine significantly decreased serum glucose level on day 7 and 21, although they remained to be higher than those of db/m mice.

### Both administration of selenomethionine alone and in combination with serine decreased liver and fat weight in db/db diabetic mice

**FIGURE 2:**
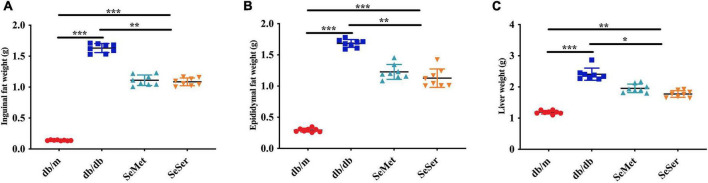
Administration of selenomethionine and serine decreased liver and fat weight in db/db diabetic mice. **(A)** Inguinal fat weight; **(B)** epididymal fat weight; **(C)** liver weight. Values are expressed as mean ± SEM, *n* = 8. *P < 0.05, **P < 0.01, ***P < 0.001.

As showed in Figure 2, mice in the SeMet and SeSer groups had significantly lower liver weight, inguinal and epididymal fat weight than those in the db/db group, however, the weight of liver, inguinal and epididymal fat of mice in the SeMet and SeSer groups remained to be significantly higher than those of mice in the db/m group.

### Both administration of selenomethionine alone and in combination with serine increased alpha-diversity of gut microbiota in db/db diabetic mice

**FIGURE 3:**
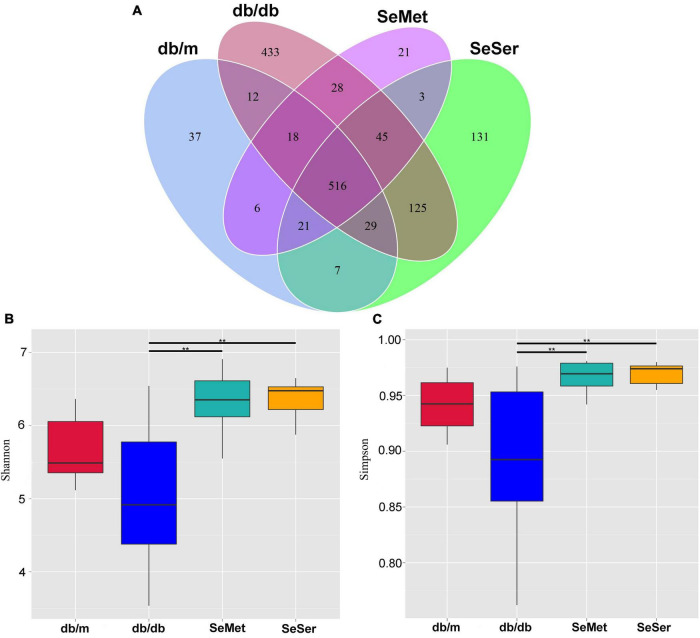
Administration of selenomethionine and serine increased alpha-diversity of gut microbiota in db/db diabetic mice. **(A)** Venn diagram of OTUs; **(B)** Shannon index; **(C)** Simpson index. Values are expressed as mean ± SEM, *n* = 8. **P < 0.01.

We compared the microbial population in the feces of mice from the four treatment groups by using the 16S rDNA phylogenetic approach. The Venn diagram results showed that 516 OTUs were universal to all treatment groups among a total of 1432 detected OTUs (Figure 3A). There were 37 unique OTUs in the db/m group, 433 unique OTUs in the db/db group, 21 unique OTUs in the SeMet group and 131 unique OTUs in the SeSer group. The α-diversity, as indicated by the Simpson and Shannon index, was significantly higher in mice in the SeMet and SeSer groups than those in the db/db group, whereas no significant difference was observed among the SeMet, SeSer and db/m group (Figures 3B,C).

### Both administration of selenomethionine alone and in combination with serine altered microbiota composition in db/db diabetic mice

**FIGURE 4:**
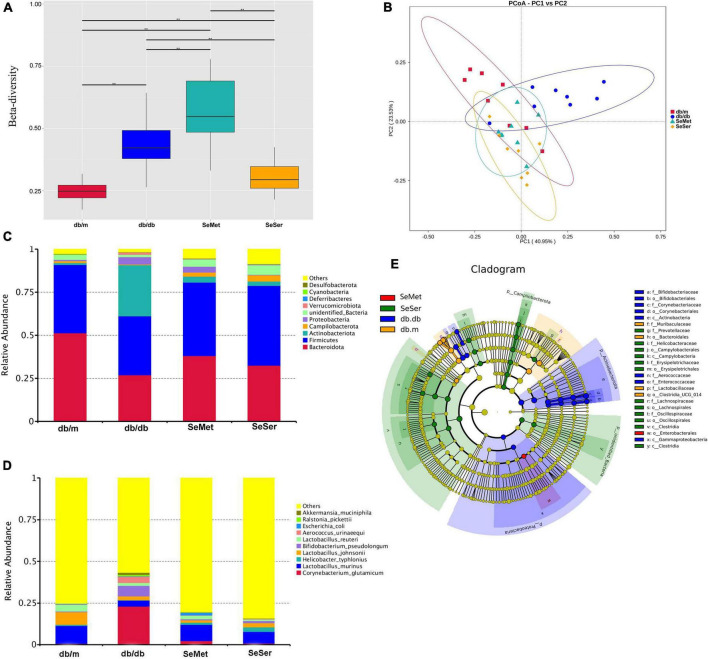
Administration of selenomethionine and serine altered microbiota composition in db/db diabetic mice. **(A)** Beta-diversity at the species-level; **(B)** PCoA plot of themicrobiota based on a weighted UniFrac metric; **(C)** relative abundance of predominant bacteria at the phylum level; **(D)** relative abundance of predominant bacteria at the species level; **(E)** taxonomic cladogram generated from LEfSe of metagenomic sequencing data. Values are expressed as mean ± SEM, *n* = 8. *P < 0.05, **P < 0.01.

The beta diversity at the species-level in mice in the four treatment groups were significantly different from each other (Figure 4A). Moreover, the results of the unweighted Unifrac PCoA analysis showed that the microbial structure in the feces of mice from db/db group was separated from the other three groups (Figure 4B). At the phylum level, mice in the db/db group had decreased abundance of Bacteroidetes and Firmicutes and increased abundance of Actinobacteriota, when compared with those in the db/m group; Administration of selenomethionine alone and in combination with serine retrieved these changes (Figure 4C). At the species level, mice in the db/db group had increased abundance of *Corynebacterium glutamicum*, *Bifidobacterium pseudolongum*, and *Aerococcus urinaeequi*, and decreased abundance of *Lactobacillus murinus* and *Lactobacillus johnsonii*; Administration of selenomethionine alone and in combination with serine restored these changes (Figure 4D). Analysis with the linear discriminant analysis (LDA) effect size (LEfSe) method showed that the db/db group was characterized by Actinobacteriota, while the SeMet group was characterized by Enterobacterales and the SeSer group was characterized by Campilobacterota (Figure 4E).

**FIGURE 5:**
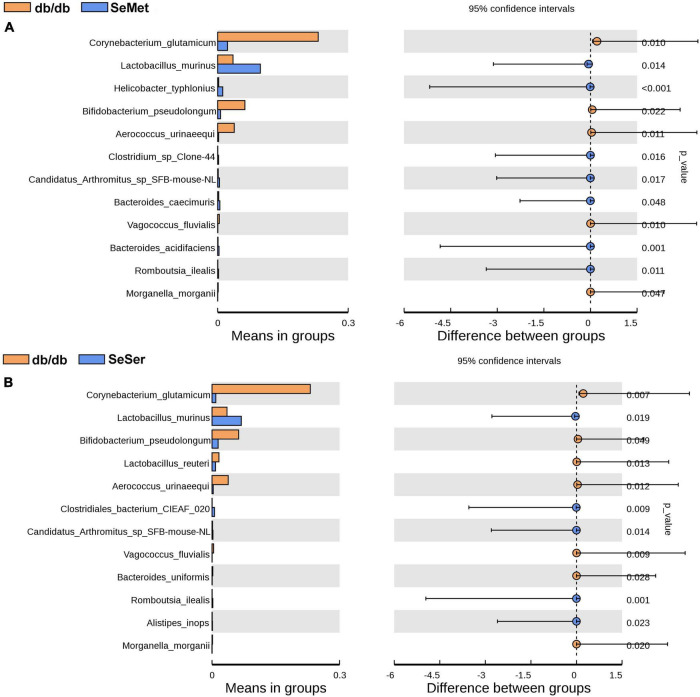
The difference of microbe abundance in the db/db, SeMet and SeSer groups by using T-test analysis. **(A)** Difference between the db/db and SeMet group; **(B)** difference between the db/db and SeSer group.

We further analyzed the difference of microbe abundance in the db/db, SeMet and SeSer groups by using T-test analysis. As shown in Figure 5, the results showed that *C. glutamicum*, *B. pseudolongum*, and *A. urinaeequi* were significantly decreased, whereas *L. murinus* was increased in mice in the SeMet and SeSer groups, when compared with those in the db/db group.

### Fecal microbiota transplantation decreased body weight and glucose level in db/db diabetic mice

**FIGURE 6:**
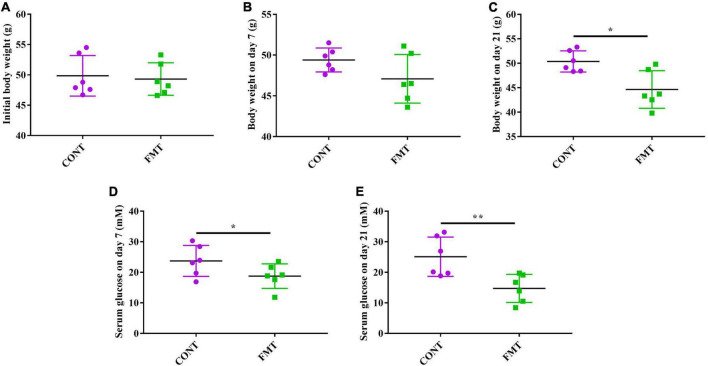
FMT decreased body weight and glucose level in db/db diabetic mice. **(A)** Initial body weight; **(B)** body weight on day 7; **(C)** Body weight on day 21; **(D)** serum glucose level on day 7; **(E)** serum glucose level on day 21. Values are expressed as mean ± SEM, *n* = 6. *P < 0.05, **P < 0.01.

The db/db mice in the CONT and FMT group had similar body weight at the start of the experiment (Figure 6A), while we observed no significant changes in body weight after FMT for 7 days (Figure 6B). However, db/db mice in the FMT group had significantly decreased body weight on day 21, when compared with those in the CONT group (Figure 6C). Moreover, db/db mice in the FMT group had significantly decreased glucose level on day 7 (Figure 6D) and day 21 (Figure 6E) when compared with those in the CONT group.

## Discussion

Selenium has been proved to lower blood glucose level in several rodent models. In STZ-induced diabetic rats, dietary supplemented with inorganic selenium for 24 weeks significantly lowered serum glucose concentration (Becker et al., 1996). Additionally, dietary selenium improved glucose intolerance in high-fat diet-fed mice (Wang et al., 2022). However, diabetic rats also showed resistance to selenium, as sodium selenite only decreased glucose concentration for the first two weeks, while they had no further effects for the last four weeks of the experiment (McNeill et al., 1991). Importantly, dietary supplemented with high dose of different types of selenium may exert contrary effects. For instance, selenite can alleviate hyperglycemia *via* serving as an insulin mimic, whereas selenite inhibit insulin signaling in diabetic mice (Pinto et al., 2011; Steinbrenner, 2013; Zhou et al., 2015). Selenium compounds are classified as organic and inorganic. Compared with inorganic selenium, organic selenium is proved to be more easily absorbed and with better biocompatibility. In the present study, we explored the effects of selenomethionine, organic selenium, on glucose homeostasis by using the db/db diabetic mice. According to our results, the beneficial effects of selenomethionine on the control of glucose level were further confirmed. Moreover, the body weight and adipose tissue weight were both decreased in diabetic mice after the administration of selenomethionine. These results suggested that selenomethionine could be potentially used for the treatment of diabetes.

Serine has been proved to exert many beneficial effects including maintaining oxidative status and alleviating inflammatory response in several animal models (Zhang et al., 2018; Zhou et al., 2021). Particularly, serine improved glucose tolerance and insulin sensitivity in high-fat diet-fed mice (Zhou et al., 2018). However, it did not significantly lower body weight and adipose weight/body weight ratio. Recently, a study showed the synergistic effect of serine and selenocompounds on glutathione peroxidase concentration (Wang et al., 2016), which indicated that serine could be a potential candidate for combined selenium administration. We further explored the combined effects of serine and selenium on glucose level in db/db diabetic mice, and the results showed that administration of selenomethionine in combination with serine significantly decreased body weight and glucose level. Notably, they showed better effects than administration of selenomethionine alone, although the difference did not reach significance. Our results further confirmed the synergistic effect of serine and selenocompounds on maintaining the glucose homeostasis in diabetic mice.

For decades, increasing evidences suggested that gut microbiota play critical roles in type 2 diabetes. As an essential micronutrient, selenium can play critical roles in oxidative stress and immune function through modulating gut microbiota composition (Ferreira et al., 2021). Serine can also regulate the composition of gut microbiota and alleviated inflammation (Zhang et al., 2018). However, whether selenomethionine and serine can improve the microbial composition in diabetic mice were not previously explored. Our results suggested that selenomethionine and serine restored alpha and beta diversity which were decreased in db/db diabetic mice. Furthermore, selenomethionine and serine retrieved the abundance of microbiota composition. Notably, administration of selenomethionine in combination with serine increased the abundance of *L. murinus* and *L. reuteri*, which are generally considered to be beneficial microbes (Goldstein et al., 2015). FMT, namely stool transplantation, is an approach that transplants feces from a donor into another recipient to directly change the intestinal microbiota of the recipient to restore the composition, in order to gain beneficial treatment effects (Wang et al., 2019). To confirm that microbes mediate the beneficial effect of selenomethionine and serine on glucose homeostasis in diabetic mice, we further transplanted the freshly collected feces from mice treated with selenomethionine and serine to db/db diabetic mice. We found that db/db diabetic mice had decreased body weight and serum glucose level after FMT, which further proved that selenomethionine and serine alleviated hyperglycemia *via* modulating gut microbiota.

## Conclusion

In conclusion, our results suggested that administration of selenomethionine decreased body weight, adipose tissue weight and serum glucose level in db/db diabetic mice. Importantly, administration of selenomethionine in combination with serine exerted better effects than selenomethionine alone. Furthermore, a combined administration of selenomethionine and serine restored the microbial composition in diabetic mice. FMT decreased body weight and glucose concentration in db/db mice, further indicating that microbes play critical roles in the beneficial effects of selenomethionine and serine. Thus, we concluded that administration of selenomethionine in combination with serine benefits diabetes via gut microbes.

## Data availability statement

The data presented in the study are deposited in the NCBI SRA database repository, accession number is PRJNA880689.

## Ethics statement

The animal study was reviewed and approved by the Animal Care and Use Committee of China Agricultural University, and conformed to the Guide for the Care and Use of Laboratory Animals.

## Author contributions

XC and YY designed the experiments and contributed to the literature search, interpretation, writing, and proofreading of the manuscript. JC performed the animal experiments. XC and JC analyzed the 16S rRNA data. All authors approved and contributed the submitted version.
